# Human Adenovirus Associated Hepatic Injury

**DOI:** 10.3389/fpubh.2022.878161

**Published:** 2022-04-28

**Authors:** Nan Zheng, Yan Wang, Hechen Rong, Kun Wang, Xiaoping Huang

**Affiliations:** ^1^Department of Infectious Diseases, First Affiliated Hospital of Soochow University, Suzhou, China; ^2^Department of Gastroenterology, First Affiliated Hospital of Soochow University, Suzhou, China

**Keywords:** human adenovirus, hepatitis, pathogenesis, pathology, therapy

## Abstract

Human adenovirus (HAdV) is a common virus, but the infections it causes are relatively uncommon. At the same time, the methods for the detection of HAdV are varied, among which viral culture is still the gold standard. HAdV infection is usually self-limited but can also cause clinically symptomatic in lots of organs and tissues, of which human adenovirus pneumonia is the most common. In contrast, human adenovirus hepatitis is rarely reported. However, HAdV hepatitis has a high fatality rate once it occurs, especially in immunocompromised patients. Although human adenovirus hepatitis has some pathological and imaging features, its clinical symptoms are not typical. Therefore, HAdV hepatitis is not easy to be found in the clinic. There are kinds of treatments to treat this disease, but few are absolutely effective. In view of the above reasons, HAdV hepatitis is a disease that is difficult to be found in time. We reviewed and summarized the previously reported cases, hoping to bring some relatively common characteristics to clinicians, so as to facilitate early detection, early diagnosis, and early treatment of patients.

## Introduction

Human adenoviruses (HAdVs), the widespread pathogens of the Adenoviridae family, are non-enveloped, double-stranded deoxyribonucleic acid (DNA) viruses. About 60 years earlier, HAdV had been independently discovered by Rowe et al. and written by Hilleman et al. (1, 2).

Human adenovirus possesses a similar icosahedral capsid with a diameter of approximately 90 nm and contains the trimeric fibrin with variable length (3, 4). The capsid is wrapped in a tightly packed viral protein and DNA genome (between 34 and 37 kbp), known as core (5, 6). HAdV is divided into serotypes (1–104) together with single species (A-G) (Table 1; http://hadvwg.gmu.edu/) through conventional serotype, viral DNA restriction enzyme digestion, sequencing, and the serotype-specific PCR.

**Table 1:** Current spectrum of known HAdVs.

A	12, 18, 31, 61
B	3, 7, 11, 14, 16, 21, 34, 35, 50, 55, 66, 68, 76–79
C	1, 2, 5, 6, 57, 89, 104
D	8–10, 13, 15, 17, 19, 20, 22–30, 32, 33, 36–39, 42–49, 51, 53, 54, 56, 58, 59, 60, 63, 64, 65, 67, 69–75, 80–88, 90–103
E	4
F	40, 41
G	52

*The HAdV species (A-G) and types (104) belonging to individual species are indicated*.

In immunocompetent individuals, HAdV infection usually causes upper respiratory, gastrointestinal (GI), or conjunctival involvement. The vast majority of cases are self-restricting, while in the immunocompetent hosts, fatal and disseminated infections are rare. HAdV can sometimes result in serious infections in the immunocompromised individuals, for example, hemorrhagic cystitis, gastroenteritis, pneumonia, encephalitis, nephritis, enterocolitis, hepatitis, or the disseminated diseases, leading to the evident incidence rate and mortality. Some serotypes can lead to more seeking infections, even to the death of immunodeficient infants and patients. For instance, HAdV7, HAdV5, and HAdV3 are the causes of acute respiratory infections, and the recently, the outbreak of HAdV7 at the rehabilitation center of New Jersey caused 11 deaths (https://www.nj.gov/health/cd/topics/adenovirus.shtml), whereas HAdV41 and HAdV40 are known to result in persistent and acute gastroenteritis in children (7).

On account of their genetic heterogeneity, HAdV species have very diverse tropism, leading to the infection of various tissues and organs. Some of HAdV species E and D (HAdV-D) mainly result in keratoconjunctivitis (EKC) (8), the infections of HAdV-C, B, and A can cause GI, respiratory, and urinary diseases, and the GI disease is also resulted from HAdV-E and F (9, 10). Subgroup D adenovirus serotype is known for its tendency to the eye, leading to epidemic EKC or conjunctivitis (11). Hepatitis is mainly correlated with subgroup C serotypes (HAdV 5, 2, and 1), especially serotype 5 infection is related to the occurrence of hepatitis in liver transplant recipients (12). In the following, we will review HAdV and HAdV hepatitis.

## Epidemiology of HAdV

Adenovirus infection can be resulted from latent virus reactivation or contact with the infected individuals. Adenovirus can be transmitted by the inhalation of infected aerosols, person-to-person contact, fecal-oral route, direct conjunctival inoculation, or contact with infected blood or tissue. Infections occur at any time of the year without apparent seasonal (12), despite the great majority of epidemics occurs in early spring or winter (13). In the meanwhile, HAdV has a worldwide distribution. The regional or local epidemics are described, but many infections occur as sporadic events. HAdV is resistant to numerous disinfectants; however, ethanol solution (95%) is a useful disinfectant (14).

Adenovirus infections are most common in the healthy children (especially children under 4 years) (15), adults in the closed environment (such as military and college), and immunocompromised patients. In immunocompetent people, HAdV make up exceed 50% of pneumonia cases and febrile respiratory illness (FRI) among the unvaccinated recruits, in the United States, simultaneously globally (16).

In immunocompromised patients, adenovirus infections are almost described in the recipients of solid organ transplantation (SOT) together with hematopoietic stem cell transplant (HSCT). In patients with human immunodeficiency virus (HIV) infection, the incidence of HAdV infection is between 12 and 28%. Among the recipients of HSCT, for the HAdV infection, its incidence is between 3 and 47%. Available data indicate that the incidence rate of allogeneic (from 5 to 47%) HSCT receptor is much greater than that of the autologous HSCT receptor (between 2.5 and 14%) (17, 18). Furthermore, the studies have shown that the adenoviral infections are more prevalent in the patients containing acute graft-versus-host disease (GVHD), severe T-cell depletion, cord-blood donors, human leukocyte antigen (HLA) mismatch, and patients who use of alemtuzumab (10). In addition, a wide variety of SOT populations have been reported to be infected with adenovirus, including those receiving liver, renal, heart, intestinal, and lung transplants. The HAdV infection incidence in the recipients of SOT is between 5 and 22%, generally within the first 6 months after the transplantation. For the adenovirus infection, its risk factors in the recipients of SOT contain liver transplant and small bowel recipients, patients receiving anti-lymphocyte antibodies, child transplant recipients, and patients with recipient negative or donor positive adenovirus status (19).

The incubation period of HAdV infection is determined by the transmission mechanism and virus serotype, between a few days and more than a week. In patients with normal immune function, the viral shedding from throat of patients with common cold will fall off for about 1–3 days; the virus shedding from throat, nose, eyes, or stool of patients with pharyngeal conjunctival fever were for 3–5 days; the virus shedding from corneal conjunctivitis eyes were for 2 weeks; the virus shedding from stool or throat of children with systemic or respiratory diseases were for 3–6 weeks. Despite the details of virus shedding in the immunocompromised patients are restricted, the shedding time is generally longer. The proposed positions of viral latency contain the intestine, pharynx (adenoids and tonsils), lymphocytes, and urinary tract, despite some of them are still controversial (20).

## High-Risk Factors of HAdV Hepatitis

Hepatitis can be caused by a variety of causes. If not treated, it can lead to liver transplantation, death, or acute liver failure (ALF). Possible causes of hepatitis contain drugs, autoimmune hepatitis, toxins, venous obstruction, ischemia, and metabolic together with infectious causes, containing cytomegalovirus (CMV), varicella zoster virus (VZV), hepatitis A to E, Epstein–Barr virus (EBV), herpes simplex virus (HSV), and human adenovirus. Although human adenovirus (HAdV) hepatitis was rarely been reported, HAdV hepatitis can occur in immunocompromised patients. First of all, we will mainly introduce the high-risk factors of HAdV hepatitis.

In a recent study, 66% of the patients were patients with pediatrics. This observation is very consistent with the outcomes of a recent meta-analysis, which found that 64% of cases of HAdV hepatitis occurred in children (21, 22). In the pediatric setting, the most prevalent predisposing condition may be liver transplantation. In the meantime, children may also develop HAdV hepatitis in the process of immunosuppression after the transplantation of hematopoietic stem cell, chemotherapy for solid malignancies or lymphoblastic leukemia, and severe combined immunodeficiency (SCID) (23).

However, some studies have shown that the proportion of HAdV hepatitis in adult patients who are immunosuppressed after the transplantation of hematopoietic stem cell is higher than that in adult patients after liver transplantation (24, 25). Adults are probably developed HAdV hepatitis when SOT other than the liver or chemotherapy leads to immunosuppression (26). All in all, in SOT recipients, most reported cases of HAdV hepatitis appeared in the recipients of liver transplant. What is more, it was reported that one patient was just given chemotherapy about a year prior to the HAdV infection. Despite he was not immunosuppressed actively, his residual lymphoplasmacytic lymphoma was found to involve kidney, spleen, and bone marrow at the autopsy, and HAdV infection was discovered in his liver. It is well known that lymphoma may cause a certain degree of immunosuppression (27). The study suggests that the immunosuppression degree resulted from lymphoma is probably enough to cause patients to develop the fatal HAdV hepatitis (22).

In conclusion, most risk factors for HAdV hepatitis include SOT, human immunodeficiency virus infection, chemotherapy, the transplantation of hematopoietic stem cell, SCID, and lymphoma (21, 28).

## Pathogenesis of HAdV Hepatitis

Despite the human adenoviral hepatitis has been reported before, it mainly appears in the manner of small series and individual case reports and does not always emphasize the pathogenesis. The infection of human adenovirus rarely leads to serious pathological changes; however, the intravenous injection of high-dose replication-deficient HAdV vector into the body of patients will lead to rapid innate immune system activation, resulting in disseminated intravascular coagulation, cytokine storm syndrome, hepatotoxicity, and thrombocytopenia. Meanwhile, HAdV-C5 vectors have high hepatic tropism, and the intravascular administration of HAdV-C5 vectors can result in acute liver toxicity. So, the pathogenesis of hepatitis caused by HAdV-C5 vectors can be partly represented the hepatitis caused by HAdV.

In the article of Alemany et al., after intravenous virus administration, virus particles can be isolated in endothelial cells of splenic sinuses and liver, tissue macrophages, and platelets *via* directly binding to the receptors of cell surface (29, 30). In addition, tissue resident macrophages in the liver, for instance, Kupffer cells, can also isolate a large number of HAdV particles (31–33). Coagulation factors that contain Gla domain can mediate the entry of virus into hepatocytes through binding to the main viral capsid protein hexon (34). These coagulation factors contain FX, FIX, and FVI, and FX has the highest affinity with the HAdV-C5 hexon (35). It had been proven that protein associated with LDL receptor and heparan sulfate proteoglycan (HSPG) can mediate the cell entry of the coagulation factor HAdV-C5 complexes existed *in vitro* (36). Nevertheless, macrophages residing in the tissues are only the first line of defense against the invading pathogens, and the activation of innate immune system sensors was triggered by these cells. Then, 10 min after the HAdV-C5 intravenous injection, in the liver, the Kupffer cells activated the transcription of proinflammatory cytokine IL-1a (33). Subsequently, IL-1a is synthesized continuously, which triggers the generation of pro-inflammatory cytokines depended on IL-1RI, containing TNF-a and IL-6, as well as chemokines CXCL2, CXCL1, and CCL2 (33). Then, IL-1a is constantly being synthesized and triggers the generation of pro-inflammatory cytokines depended on IL-1RI, containing TNF-a and IL-6, as well as chemokines CXCL2, CXCL1, and CCL2 (33). In the meantime, after the isolation of HAdV from blood, Kupffer cells undergo necrotic death within 60 min after intravenous injection of HAdV. For the death of necrotic Kupffer cell, the result is the activation of systemic inflammatory response, resulting in the release of pre-synthesized cytosolic IL-1a together with other proinflammatory mediators, containing HMGB1, in circulation (37). When necrotic cells are released, systemic and local IL-1RI signals are initiated by IL-1a (38). Thus, IL-1a activation can also cause severe immunopathology of the infected liver.

The cells of natural killer (NK) existed in a liver-directed gene transfer mouse model were found to play a major role in eliminating hepatocytes infected with the HAdV-C5-based vector (39). Within 3 days after the intravenous injection of vector, NK cells existed in the spleen were activated highly and expressed IFN-c, glutamate B, and perforin. Besides, in the cells of NK, the expression of the cytotoxic effectors is completely determined by the functional IFN-I signal. The research has suggested that on account of the lack of IFN-I signal, there is a cell population dysfunction of NK and the prolonged duration of transgenic hepatocytes infected by virus. Therefore, the removal of virus infected cells from liver is principally mediated *via* the activated cells of NK. The same research exhibits that viral infection cell lysis mediated by NK cell requires the main NK cell activation receptor NKG2D (40). NK cells mediate antiviral innate immune responses by producing cytokines and dissolving infected cells when activated (41–43). Activated NK cells help to control the early stage of infection and promote the in-depth initiation for the virus-specific immunity of CD8+ T-cell. In one study, the aggravation of hepatocellular damage observed during human adenovirus infection associated with a raising expression of IFN-γ and the CD8+ T-cell absolute numbers in liver (44). This is in accordance with other liver infection models, where the emergence of hepatocyte injury is related to the obtainment of the effector function *via* CD8+ T-cells (45).

Combined with the previous studies, the indirect effect related to immune damage is more important than direct effect of viral infection. As a recent study shows that disseminated infections caused by HAdV-C in immunocompromised hosts are known to trigger severe acute inflammatory responses, which are manifest in part by high blood levels of inflammatory cytokines and chemokines, such as IL-1, IL-6, TNF-**α**, IFN-a/b, IFN-c, and IL-8 (46). While promoting virus-infected cells to be cleared from the body, these inflammatory cytokines and chemokines aggravate virus-induced inflammation, severe immune pathology, and damage to healthy tissues, leading to multiple organ dysfunction and even death. In addition, Fejer et al. have shown that intravenous or intraperitoneal administration of HAdV-C2 or HAdV-C5 leads to the development of hypersensitivity to secondary challenge of mice with lipopolysaccharide (LPS) (47).

## Pathological Features of HAdV Hepatitis

Histopathological assessment is the gold standard for diagnosing the HAdV hepatitis. When tissues are infected with human adenovirus, routine examination of eosin-stained and hematoxylin slides can exhibit different degrees of regeneration, reactivity, and inflammation. By *in situ* and immunoperoxidase hybridization, we can confirm the presence of the virus in tissues (such as liver) (19). Human adenovirus-infected cells (which known as “smudge cells”) are a typical type of the intranuclear inclusions containing thin cytoplasmic margins and large nuclei, basophilic inclusions.

Recently, the research exhibited twelve human adenoviral hepatitis cases, containing autopsy specimens and biopsy (22). All of the above cases possessed histological evidence of liver injury and laboratory or immunohistochemical (IHC) evidence of the infection of active human adenovirus (e.g., electron microscopy, polymerase chain reaction (PCR), virus culture, and other additional approaches). The two pathologists review all of the cases (KBS and JPTH), and all the cases were discussed and agreed. They examined various morphological findings, containing granulomatous inflammation, varying degrees of necrosis, and the existence of inclusion bodies, lymphocytes, and neutrophils.

In this paper, tissue sections of all the cases revealed non-banded coagulative hepatocyte necrosis, and necrosis was divided into spotty hepatocyte necrosis, submassive necrosis, and massive necrosis, with a corresponding percentage of necrosis ranging from <5 to 95%. Punctate hepatocyte necrosis was employed for expressing the apoptosis or necrosis of isolated hepatocytes in lobules, whereas massive necrosis referred to extensive necrosis of nearly all or all normal liver lobules. The submassive necrosis was applied for expressing a moderate number of necrosis, generally manifested as confluent necrotic region. Despite this necrosis is generally completely non-banded, half of the cases confirmed raised involvement in the periportal area on the biopsy specimens. On the contrary, autopsy revealed complete non-banded necrosis. The change from extreme punctate, focal necrosis to massive, and wide necrosis seems to be determined by the progression and timing of HAdV infection as well as sampling. Notably, the percentage of necrosis in this small biopsy sample is probably underlying misleading on account of the restrictions of sampling and the liver heterogeneous involvement. Histological variations revealed that there exists some anatomical heterogeneity in liver. In a case, despite peripheral liver biopsy revealed only rare granuloma and focal portal vein lymphocyte infiltration, the biopsy of discrete liver lesions guided by simultaneous image observed *via* abdominal MRI and CT exhibited a large number of intranuclear inclusions and necrosis. A total of 33% of the patients with imaging reports provided for review suggested heterogeneous or focal imaging outcomes. Necrosis will also progress as the progresses of disease. The comparison of autopsy specimen and biopsy specimen patients indicated that the percentage of necrosis raised from 20 to 60% from the initial biopsy time.

In all cases in this paper, characteristic stains in hepatocytes to inclusion bodies in vitreous nuclei were reflected in tissue sections. These inclusions are most prevalent at the edge of the necrotic region, however, sometimes scattered in lobules. Despite these inclusions are nearly basophilic, a few cases suggest that the inclusions have glassy eosinophilic regions. Well-developed and particularly large inclusion bodies usually possess a central eosinophilic glassy appearance, and basophilic chromatin is marginalized. Besides, in a case (8%; 1/12), inclusion bodies were also found in the bile duct epithelium. Adenoviral ascending biliary hepatitis has formerly been reported to be associated with the GI infection existed in the immunosuppressed children, which may be the outcome of GI ascending infection entering biliary tract (48). Nonetheless, there was few related inflammations in most cases (58%, 7/12). If exists, inflammation is lymphoid and focal, while no neutrophilic inflammation was identified.

## Imaging Characteristic of HAdV Hepatitis

Although the imaging characteristics of HAdV hepatitis are often non-specific, radiology plays a more essential role in the suspected patients with HAdV hepatitis, it is conducive to exclude other causes. Non-specific images of magnetic resonance imaging and computed tomography revealed periportal edema and liver enlargement. The clear low attenuation regions and uneven enhancement existed on the CT, whereas periportal edema possessed high signal intensity regions on MRI T2-weighted images (49). Among some patients with HAdV hepatitis, we can also find multiple hypodense lesions or single hypodense lesion in CT.

In one case, a biopsy guided by simultaneous image of a liver lesion revealed massive necrosis, while the lesion was thought to represent an abscess when it was first found on a CT scan of the abdomen because of the lesion with an intrahepatic rim-enhancing (22). In the same study, a patient's lesion was mistaken for cirrhosis by ultrasound because of the heterogeneously necrotic appearance of the liver. Another patient was explicated *via* CT scan as a lymphoma involving the liver. In fact, these two patients were diagnosed with HAdV hepatitis. Similar heterogeneous enhancement and clear low attenuation areas have been described in the previous reports and were radiologically mistaken for metastases, other infections, or hepatolenticular degeneration (HLD) (24–26, 50). Therefore, these different imaging findings and interpretations confirm the significance of histological evaluation of liver biopsy. Simultaneously, it is significant to be familiar with the most related key radiological features and then combined with the key clinical information, which is probably afforded sufficient information to describe the characteristics of the lesion. In the end, we can also successfully differentiate HAdV hepatitis from other diseases.

## Diagnosis of HAdV

Human adenovirus can be detected through indirect or direct immunofluorescence, routine or shell bottle culture, together with the PCR of the infected sites (e.g., urine, stool, blood, swabs, nasopharyngeal aspirates, bronchoalveolar lavage, and washings) (51). The available diagnostic approaches for the infections of adenovirus [e.g., electron microscopy, culture, histopathology, detection of antibodies, direct antigen detection, PCR, immunohistochemistry, and *in situ* hybridization (ISH)] are determined by the collected samples and infection location.

Traditional or shell bottle culture is a gold standard, but it may not be sensitive to some samples (e.g., blood) and is probably take several days to induce the cytopathic effect (CPE). Adenovirus-related CPE is typically expressed as the grapevine, with irregular aggregates composed of enlarged circular cells revealing characteristic refractable intranuclear inclusions. CPE slowly develops and variations can be found within 28 days after the inoculation, most of which occur in the first culture week (12). The time of detection is determined by various factors, containing the serotypes or adenovirus subsets, viral load inoculated, culture condition, or cell lines utilized.

The electron microscopy can be employed for identifying the HAdV, particularly in stool. What is more, immunoelectron microscopy can improve the detection sensitivity. Generally speaking, although the number of enteric adenoviruses in stool is much larger than the number of isolates of respiratory adenovirus, in the stool, the existence of adenovirus cannot diagnose GI diseases. In the past, these approaches for the identification of adenovirus in the clinical specimens (especially GI specimens) were a main method, but their convention use has decreased in the past several years.

Histopathological evaluation is still a gold standard for diagnosing the diseases of invasive adenovirus. This technique for the detection of adenovirus has been described above.

Fluorescent antibody (FA) detection can be employed for the direct detection (generally from the respiratory samples), simultaneously for identifying virus isolates cultured by shell bottle method and traditional methods. Despite FA is extensively utilized, its sensitivity is restricted, especially in detecting the double infection (52).

Adenovirus nucleic acid can be determined through a variety of molecular approaches. Since the N-terminal area of hexagonal gene and trans-activated area of E1A are very conservative between serotypes, primers for these areas are generally utilized for PCR detection (53).

Polymerase chain reaction is an extensively utilized approach to detect the adenovirus through amplifying and determining virus genome, which greatly replaces the virus culture together with other old diagnostic approaches (10). Through polymerase chain reaction, we can test stool, blood, respiratory secretions, and tissue specimens. Quantitative and qualitative PCR approaches can be utilized. Simultaneously, PCR possesses high rapidity and sensitivity. A novel molecular detection approach known as immune-PCR is expected to improve the sensitivity of fecal detection owing to it eliminates the requirement for extracting the nucleic acid (54).

Immunohistochemical staining can determine the HAdV hexagonal antigen existed in the tissue (55). In ISH together with IHC technologies for detecting the adenovirus is conducive to identify the infection of HAdV in tissue sections and enhance the sensitivity as an auxiliary means of morphological examination. In the meantime, they may localize disease and provide evidence for causality (56).

Although the sensitivity of instance electron microscopy is low, the specificity is high. The sensitivity of direct antigen detection is lower than the specificity. On the contrary, the sensitivity of detection of antibodies is higher than the specificity. Combining ISH with immunohistochemistry techniques for the detection of adenoviruses can increase the sensitivity. PCR's both sensitivity and specificity are high. The sensitivity and specificity of culture and histopathology are also high, but they are more time-consuming and laborious. In conclusion, despite HAdV-positive detection may be regarded as the diagnosis of the adenovirus infection in a proper clinical environment, PCR of infection site or pathological evidence of the invasive adenovirus disease is still required to determine causality.

## Clinical Features of HAdV Infection

### Respiratory Tract Involvement

Human adenovirus makes up at least 5–10% of childhood infections and 1–7% of the adult respiratory infections (RTIs) (12). For the HAdV RTI, its characteristic symptoms consist of fever, cough, tonsillitis, and pharyngitis. Among children, GI symptoms may be present concomitantly (57). Symptoms are usually self-limiting (within 2 weeks) in immunocompetent patients, and infection can lead to the type-specific immunity. Nonetheless, pneumonia appears in 20% of infants and newborns, and death from HAdV pneumonia has been expresses in formerly healthy adults or children (58). In 10–30% of cases, immunocompromised persons may develop into severe respiratory failure and/or transmission, and mortality rate of severe HAdV pneumonia may be as high as 50% (59).

### Ocular Involvement

Manifestations of ocular HAdV infection consist of non-specific conjunctivitis, pharyngeal conjunctival fever, and EKC (60). The most prevalent species related to the ocular mucosa acute infection is HAdV-D, which causes the clinical ocular pathogenesis, whereas HAdV-D is reported to be related to EKC outbreak, which may induce vision loss, recurrent, or chronic keratitis. David Dyer, Xiaohong Zhou, Ashrafali Mohamed Ismail, James Chodosh, Jaya Rajaiya, and Donald Seto expressed the clinical pathology for this disease and the virus molecular phenotype inducing the epidemic EKC (8). What is more, conjunctivitis occurs predominantly with HAdV-B (61).

### GI Infection

Acute HAdV infections of the GI tract occur predominantly with HAdV-F (notably HAdV-40 and−41) and can cause a lot of symptoms, containing hemorrhagic colitis, diarrhea, gastroenteritis, pancreatitis, cholecystitis, and hepatitis (62). Even if the major position of the infection is respiratory tract, HAdV infections can also cause GI symptoms, particularly in young children (15). The researches have indicated that most invasive infections seem to be induced *via* the reactivation of virus. Adenovirus persists in children's GI tract. Intestinal epithelium is the major position of virus replication, which precedes the invasion event. As a result, regular monitoring of the existence and load of adenovirus in the fecal samples has become the main diagnostic approach of patient monitoring. However, the site of reactivation in the adult setting is unclear. Hence, peripheral blood sample monitoring is a conversion method for the adult patients.

### Urinary Tract Infection

In patients undergoing HSCT and SOT, HAdV can induce urinary tract infections (UTIs). Its classical manifestations contain hematuria, dysuria, the dysfunction of renal transplantation and hemorrhagic cystitis (HC) (63). Despite there have been reports of dialysis-dependent or fatal renal failure, obstructive uropathy, necrotizing tubulointerstitial nephritis, and fatal dissemination, HAdV UTIs usually abate spontaneously. Most common species associated with HC is HAdV-B, including HAdV-11, -34, -35, -3, -7, and -21 (64). Diagnosis can be confirmed through culture or PCR in serology or urine, and the viral infection of the renal tubular epithelial cells containing intranuclear inclusions and stained cells was demonstrated by renal biopsy.

### Liver Infection

We almost reviewed all case reports in the recent 10 years and summarized the liver function index of these patients (22, 23, 26, 65–75). The average of ALT was 1,607 U/L, with a range from 87 to 9,150 U/L. The average of AST was 4,671 U/L, with a range from 76 to 16,050 U/L. The average of TBIL was 6.2 mg/dL, with a range from 0.4 to 31.3 mg/dL. The average of ALP was 820 U/L, with a range from 123 to 2,802 U/L. The average of γ-glutamyltransferase was 790 U/L, with a range from 98 to 1,563 U/L. In a case report written by Kawashima et al., their observation raises the possibility that elevated γ-glutamyltransferase could be a sentinel marker for HAdV hepatitis. In subsequent studies by Onda et al., they also suggest that an increase in γGTP (100 IU/L or more) appears more than 2 weeks before the onset of hepatitis may be useful for the early diagnosis of HAdV hepatitis. All in all, adenovirus hepatitis is more common in children, the elderly, and adults with compromised immune systems than other types of hepatitis, and its onset is more acute. In other viral hepatitis, except viral hepatitis A and E often cause acute hepatitis, viral hepatitis B, C, and D usually cause chronic hepatitis. On the laboratory examination, adenovirus hepatitis shows markedly elevated liver enzymes, which should be differentiated from drug-induced liver injury. In addition, the other clinic features of HAdV hepatitis will be described below.

### Other Disease

Despite 10–30% of HSCT recipients develop the disseminated disease with the infection of HAdV, in the immunocompetent hosts, disseminated infection of HAdV is rare (76). At the same time, rare manifestations for the infection of HAdV contain cardiomyopathy and myocarditis, meningitis, encephalitis, pulmonary dysplasia, mononucleosis-like syndrome, sudden infant death, and intussusception in children (77).

## Therapy of HAdV Hepatitis

The first and most significant treatment is still the reduced immunosuppression and supportive care. Nevertheless, there do not exist consensus on which immunosuppressive drugs to stop or decrease and the time to restart immunosuppression. Remission of viremia after reduction of immunosuppression shows the significance of the immune recovery during infection (78, 79). On the other hand, it is difficult to identify the primary cause that is on account of an increase in antiviral therapy and a decrease in immunosuppression or the combination of these treatments in the resolution of the disease (80).

Timely initiation of antiviral therapy is necessary to prevent or successfully control human adenovirus hepatitis. The currently available immunotherapy and agents against HAdV based on the nucleoside analogs are not supported through the randomized prospective clinical experiment of brincidofovir and cidofovir (cidofovir's lipid-conjugated prodrug) that seem to be useful for treating progressive, disseminated and severe human adenovirus diseases, which include HAdV hepatitis.

Despite cidofovir is a cytosine nucleotide analog, which suppresses the viral DNA polymerase, has been reported that its efficacy depends partly on patients' partial immune recovery, the most commonly used antiviral medicant is still cidofovir. Meanwhile, cidofovir has main side effects, including severe nephrotoxicity and neutropenia. So, the side effects of cidofovir limit its wide application in clinic.

In comparison with cidofovir, brincidofovir has less renal toxicity and has better oral biological availability and enables administration once or two times a week. Although brincidofovir also has some toxic side effects, such as GI symptoms, it is the most effective anti-adenoviral medicant. The recent studies used the lipid conjugate of cidofovir to give a preemptive treatment in patients with HAdV and reveal promising outcomes, namely, the drug allows a considerable proportion of patients to clear HAdV, even without the reconstitution of immune (81, 82). A few reports have emphasized the life-saving potentiality of this drug for the immunocompromised patients (83, 84).

Ribavirin is a type of nucleoside analog that appears to possess an antiviral activity only against HAdV-c (serotypes 6, 5, 2, and 1) (85). However, the significant anti-adenovirus activity of ribavirin in patients is uncertain, and its main side effect is anemia. All in all, ribavirin is not recommended to treat serious HAdV infections. Ganciclovir is a common antiviral drug, but it needs the phosphorylation to reach the active condition. Nevertheless, human adenovirus is unreasonable to treat adenovirus infection owing to the lack of thymidine kinase (86). The efficacy of other commonly used drugs, which include foscarnet and vidarabine, in treating HAdV hepatitis is highly questionable.

Besides using antiviral medicant, intravenous immunoglobulin may be beneficial, especially in patients with hypogammaglobulinemia. In addition to the treatments described above, immunotherapy (for instance the adoptive transfer for the treatment of HAdV-specific T cells) has been employed for exceeding 10 years to combat invasive HAdV infections that do not respond to virostatic drugs (87). This is an adoptive immunotherapy that could result in a polyclonal T-cell graft, containing enriched numbers of HAdV-specific CD4+ and CD8+ T cells. Additionally, it would be better that making a transfer of specific T cells before the onset of symptoms and increasing viral load. The potential side effect of adoptive T-cell immunotherapy is GVHD. Because the isolation procedure is an enrichment of virus-specific T cells and it may result in a mixed population with a potential contamination of alloreactive T cells, which carry the risk of GVHD induction and aggravation of viral infections. Several studies have suggested that the existence of HAdV-specific T cells and immune reconstitution are very significant to clear the infection of HAdV, which gives a foundation for the treatment of human adenovirus infection (88–90). The initial studies had shown that timely initiation for the treatment is especially significant for controlling the infection of HAdV successfully. Proper immune cells from various sources have been investigated (87). Except for a variety of methods of separating HAdV-specific T cells from their respective SCT donors, other sources of virus-specific T cells were employed, containing T-cell line libraries constructed from healthy donors revealing prevalent HLA polymorphisms or partial HLA-matched third-party donors (87, 91–95). The clear link between successful virus control and timely immunotherapy initiation supports the concept of affording an off-the-shelf supply of HAdV-specific T cells (96, 97). Simultaneously, in the recipients of child SCT, the average time span between the first onset of adenoviremia and HAdV amplification exceeding critical threshold in continuous stool samples was as long as 11 days (98). During this time, separation and amplification of Don-induced HAdV-specific T cells can also be adoptive transferred after or before viremia (92). Specifically, in high-risk groups, it can be imagined to produce the virus-specific T cells prior to allogeneic SCT to guarantee access to the targeted immunotherapy timely. In conclusion, immunotherapy is an important part of combating HAdV infection in immunocompromised patients because of the current limitations of antiviral drugs.

## Summary of HAdV Hepatitis Case Report

In 2014, Ronan et al. published a review on the adenoviral hepatitis clinical characteristics, containing the follow-up reports as of July 2021. A number of 107 adenoviral hepatitis cases have been reported in the literature (21–23, 26, 65–74). Next, we will summarize these cases briefly.

Of the 107 confirmed HAdV hepatitis cases, the great majority of the patients were children under the age of 18, and the proportion was 61% (Table 2). In contrast, 42 were adults. There were slightly more male patients than female patients. In our study, the recipients of liver transplant and bone marrow transplant are 48 and 27 cases, respectively, 15 cases received chemotherapy recently, and five cases were patients with serious combined immunodeficiency. Compared with the Ronan's article, the number of patients receiving bone marrow transplant has increased, probably because of hematology science is now better developed. HAdV hepatitis caused by other diseases does not change significantly from the past, except for the addition of one patient who was underlying chronic lymphocytic leukemia (CLL). Diagnosis of HAdV hepatitis was made by patients' liver biopsy or on autopsy. The method used to identify adenovirus has been described above. Among 107 patients, the most common method was immunohistochemistry in the liver tissue. In the meantime, viral culture was also commonly used. The application ratio of these two methods was 62 and 61%, respectively, while the application proportion of electron microscopy was 45%. Comparatively speaking, PCR and ISH were relatively less used. Liver necrosis is the most prevalent histopathological manifestation. The stain cells and viral inclusion bodies could also be found in liver histopathology. Additionally, the initial symptom was fever in 84 patients. Other prevalent symptoms when present contained discomfort or drowsiness, jaundice, and diarrhea. In some situations, alkaline phosphatase, alanine aminotransferase (ALT), and aspartate aminotransferase (AST) also rapidly increased. Abdominal CT scan revealed that 15 of the 19 patients had multiple low-density lesions, while a single hypodense lesion was found in 3 of these patients. However, few positive findings were in CT imaging, suggesting the concealed of HAdV hepatitis.

**Table 2:** Characteristics of 107 patients with HAdV hepatitis.

**Basic conditions**	**Number of patients**	**Proportion of patients**
**Age**		
Pediatric (0–17)	65	61%
Adult (≥18)	42	39%
**Gender**		
Male	42	54%
Female	36	46%
**Underlying condition**		
Liver transplant	48	45%
Bone marrow transplant	27	25%
Chemotherapy	15	14%
SCID	5	4%
HIV infection	4	4%
Renal transplant	2	2%
Heart transplant	2	2%
Neonates (no known comorbidity)	2	2%
CLL	2	2%
**Method of adenovirus detection in the liver (*****N*** **=** **107)**		
Immunohistochemistry	66	62%
Culture	65	61%
Electron microscopy	48	45%
Polymerase chain reaction	16	15%
*In situ* hybridization	4	4%
**Liver histopathology (*****N*** **=** **82)**		
Necrosis	72	88%
Intranuclear inclusions	55	67%
Smudge cells	21	26%
**Presenting symptoms (*****N*** **=** **92)**		
Fever	84	91%
Lethargy/malaise	22	24%
Diarrhea	13	14%
Jaundice	8	9%
**CT imaging findings (*****N*** **=** **19)**		
Multiple hypodense lesions	15	79%
Single hypodense lesion	3	16%
Normal	1	5%
**Outcome (*****N*** **=** **107)**		
Survival	30	28%
Death	77	72%
**Treatment (*****N*** **=** **59)**	**Number of patients**	**Survival**
Reduced immunosuppression	27	15
Reduced immunosuppression + antiviral	9 cidofovir	7
	2 ribavirin	1
Liver re-transplantation	12	6
Cidofovir + IVIG	3	2
IVIG alone	4	0
Cidofovir alone	2	0

In 107 cases, only 59 patients' treatments were accurately described, while the most useful treatment was reduced immunosuppression (Table 2). Of the 27 patients, the only improvement of treatment was the decrease of immunosuppression, of which 15 survived. A number of eight other patients with antiviral drugs (ribavirin or cidofovir) and immunosuppressants also survived. Among the 12 patients, 6 were successful in liver transplantation. Intravenous immunoglobulin therapy alone or cidofovir therapy alone was unsuccessful in all 6 patients of HAdV hepatitis, while 2 of 3 patients survived using IVIG and cidofovir together. It was unfortunate that the overall prognosis for patients with hepatitis HAdV is poor, with only 30 of 107 published cases (28%) surviving. In conclusion, the great majority of HAdV hepatitis cases are fatal. The hepatitis of HAdV has few characteristic signs and symptoms, but it is generally hard to diagnose and delayed treatment is not uncommon.

## Conclusions

Among viral infections, human adenovirus infection is relatively rare, but HAdV infection is also of great clinical significance. Among immunocompetent patients, HAdV infection usually causes upper respiratory, GI, or conjunctival involvement, and the most cases are self-limited. In contrast, disseminated and severe infections (e.g., pneumonitis, gastroenteritis, hemorrhagic cystitis, nephritis, encephalitis, and enterocolitis) are more common among immunocompetent patients. However, HAdV hepatitis is uncommon, the common causes of hepatitis contain drugs, venous obstruction, ischemia, autoimmune hepatitis, toxins, and metabolic cause together with infectious causes, involving CMV, VZV, hepatitis A-E, EBV, and HSV. In our review, we mainly introduced human adenovirus hepatitis. And we summarize some characteristics of HAdV hepatitis (Table 3). The high-risk factors of HAdV hepatitis include SOT, the virus infection of human immunodeficiency, chemotherapy, the transplantation of hematopoietic stem cell, SCID, and lymphoma. The review of reported cases with HAdV hepatitis shows that the most common risk factor is liver transplantation. With the development of hematology science, the number of patients receiving bone marrow transplant has increased, and the proportion of this factor is also increasing. There are few studies on the pathogenesis of human adenovirus hepatitis, but they mainly focus on the study of adenovirus vector-induced liver disease. According to the current reports, we found that NK cells and CD8+ T cells play a major effect in the elimination of hepatocytes that infected with vector based on HAdV-C5. We expect that there are more innate and adaptive immunity studies related to HAdV hepatitis in the future. Now, the available diagnostic methods of HAdV include culture, electron microscopy, histopathology, detection of antibodies, direct antigen detection, PCR, immunohistochemistry, and ISH. Surprisingly, there is an even newer detection method, next-generation sequencing, which can help to type adenoviruses. In an appropriate clinical setting, a positive next-generation sequencing (NGS) test can be considered a diagnosis of adenovirus infection. So far, few cases that used NGS to detect HAdV have been reported, and the effectiveness of this method remains to be observed. Histopathologic assessment is a gold standard for diagnosing the HAdV hepatitis, and the histopathologic features include hepatic necrosis, viral inclusions, and smudge cells. For the HAdV hepatitis, its imaging characteristics are generally non-specific, but radiology exerts a more significant effect in suspected patients with HAdV hepatitis and is conducive to eliminate other causes. In our review, we can find multiple hypodense lesions or single hypodense lesion in CT among some patients with HAdV hepatitis. However, the positive findings are still relatively rare and are still not supported by large sample data. In terms of treatment, despite the presently available anti-HAdV drugs based on the nucleoside analogs are not supported through the prospective randomized clinical trials, antiviral medicant is partly useful. Besides, intravenous immunoglobulin and immunotherapy (e.g., adoptive transfer of HAdV-specific T-cell therapy) may be beneficial. More importantly, reducing immunosuppression is necessary. In the end, 107 human adenovirus hepatitis cases reported in the literature were gathered. We find that the clinical symptoms of HAdV hepatitis are not typical, and the most common symptom is fever. Other symptoms include lethargy or malaise, diarrhea, and jaundice. In some situations, alkaline phosphatase, ALT, and AST also rapidly increased. Generally speaking, HAdV hepatitis possesses few characteristic signs and symptoms, but it is often hard to diagnose and delayed treatment is not uncommon. So, HAdV hepatitis is usually fatal. We expect to collect further information on large samples to find clinical features of human adenovirus hepatitis in the next study.

**Table 3:** Characteristics of HAdV hepatitis.

The route of infection	Inhalation of infected aerosols Person-to-person contact Fecal-oral route Direct conjunctival inoculation Contact with infected blood or tissue
Methods of detection	Instance electron microscopy Culture Histopathology Detection of antibodies Direct antigen detection Polymerase chain reaction Immunohistochemistry *In situ* hybridization
Treatment	Reduced immunosuppression Reduced immunosuppression + antiviral drug (cidofovir/ ribavirin) Liver re-transplantation Cidofovir + IVIG IVIG alone Cidofovir alone Brincidofovir Adoptive transfer of HAdV-specific T cell therapy

## Author Contributions

NZ and XH designed the concept of the study. NZ, XH, and YW wrote the first draft of the manuscript. HR and KW edited the subsequent versions of the manuscript. All authors contributed to the article and approved the submitted version.

## Funding

The Chinese Foundation for Hepatitis Prevention and Control (31010303010946, TGQB20180371) sponsored this study.

## Conflict of Interest

The authors declare that the research was conducted in the absence of any commercial or financial relationships that could be construed as a potential conflict of interest.

## Publisher's Note

All claims expressed in this article are solely those of the authors and do not necessarily represent those of their affiliated organizations, or those of the publisher, the editors and the reviewers. Any product that may be evaluated in this article, or claim that may be made by its manufacturer, is not guaranteed or endorsed by the publisher.
